# Sex differences in rheumatoid arthritis: more than meets the eye...

**DOI:** 10.1186/1741-7015-7-12

**Published:** 2009-03-30

**Authors:** Ronald F van Vollenhoven

**Affiliations:** 1 Department of Rheumatology, Karolinska University Hospital, 17176 Stockholm, Sweden

## Abstract

Sex differences in the prevalence of autoimmune diseases such as rheumatoid arthritis (RA) are well described, but the literature is not as clear about sex differences in RA disease *course *and *prognosis*. A recent study from a very large cross-sectional international cohort demonstrated slightly worse levels of disease activity and function in female patients with RA, compared with men. These findings are discussed in the context of our evolving knowledge of sex differences in the expression of this prototypic autoimmune disease, both in terms of the actual disease activity level, the effects that the disease has on physical function, and our ability accurately to measure these aspects.

## Commentary

Many autoimmune diseases display a striking imbalance between the sexes, with females representing the majority of cases. Thus, autoimmune thyroid diseases, multiple sclerosis, and many of the rheumatological systemic autoimmune diseases such as systemic lupus erythematosus and Sjögren's syndrome affect women more often than men (Table 1, [1-6]). This is also true for rheumatoid arthritis (RA), where the sex ratio is typically around 3:1 [7]. The reasons for this overrepresentation of women are not clear, but genetic (X-linked) factors and hormonal aspects are likely to be involved [8-13].

**Table 1:** Sex ratio in various rheumatological diseases.

Disease	Female:male ratio
Sjögren's syndrome	9:1 [1]

Systemic lupus erythematosus	7:1 [2]

Rheumatoid arthritis	3:1 [3]

Systemic sclerosis	3:1 [4]

Psoriatic arthritis	1:1 [5]

Ankylosing spondylitis	1:3 [6]

**Figure 1:**
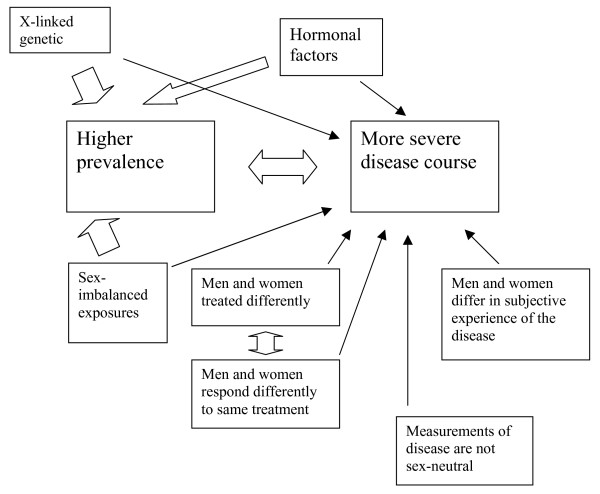
**X-linked genetic factors, hormonal factors, and exposures that may be different for men and women could all influence the prevalence of autoimmune diseases as well as their severity**. The latter aspect is, however, also influenced by many more factors, including differences in the treatments given, the response to treatments, the subjective experience of the disease, and the instruments used to measure the disease. The thick arrows indicate established associations and the thin arrows putative associations.

RA is a relatively common chronic autoimmune inflammatory disease that affects the synovial joints, and with time causes significant functional losses due to persistent inflammatory activity in the joints, destruction of bone and cartilage, and extra-articular disease manifestations. In addition to an imbalance in the prevalence as noted above, RA may also have an imbalance in disease *course *and *prognosis*. Many observational studies have suggested that, on the whole, women with RA do worse than men with the disease [14-18]. However, this issue is not as straightforward as it may seem. First of all, the observational data cited are not all in agreement, and some studies have not supported them. Second, because of the very long follow-up times required, such data are always subject to various biases. Third, it is not entirely clear what kind of 'doing worse' is most relevant. For instance, the fact that functional outcomes are worse in female patients may be attributed to the fact that greater muscle strength in men allows them to compensate in a more successful manner for functional losses, and not for differences in the disease *per se*. Finally, if the disease course in women is, indeed, more grave than in men, then the question is raised whether this is due to a) an inherent difference in the biology of the disease; b) a difference in the manner in which men and women respond to therapy; or c) the therapies given to men and women with the disease (Figure 1).

Recently, Sokka et al [19] published an analysis based on an exceptionally large international cohort of RA patients, the QUEST-RA study. This cohort of more than 6000 patients, recorded at 70 sites in 25 participating countries, was cross-sectionally analyzed with respect to demographics and disease activity parameters. The authors analyzed the 'core set' disease activity parameters for RA, an internationally agreed-upon set of seven measurements considered highly relevant for the assessment of RA [20]. These include the swollen joint count (SJC): the number of joints that are objectively swollen as determined by the examining physician, out of a prespecified number of joints, usually 68 in the case of clinical trials, but reduced to a more manageable 28 in the case of observational studies, as was done here. The SJC is generally perceived as a very 'objective' measure of RA disease activity. Other core set measures include:

a) the tender joint count (TJC) where joints are counted that are tender to palpation;

b) the patient's assessment of global disease activity on a visual analogue scale (VAS);

c) the patient's assessment of pain by VAS – these latter three outcomes are more obviously 'subjective' in nature;

d) the physician's assessment of disease activity, also usually done by VAS, and sometimes considered the gold standard;

e) an acute-phase reactant, either the C-reactive protein or the erythrocyte sedimentation rate;

f) the health-assessment questionnaire disability index (HAQ), which is not truly a measure of disease activity but of function – which can be influenced by disease activity but also by permanent damage.

A combination of four out of these seven core-set measures allows calculation of the disease activity score (DAS, or DAS28 if SJC and TJC are based on 28 joints) which gives a summary in one single number [21,22]. Finally, DAS-based cut-offs can be used to define patients considered to be in remission [23,24].

In the QUEST-RA study by Sokka et al [19], it was found that all core-set measures were higher in the female patients (representing 79% of the patients) than in the male ones, in apparent support of the thesis that female patients with RA 'do worse'. However, when calculating the differences as *effect sizes*, that is, asking the question how much of a difference in outcome could be attributed to the sex difference, rather small estimates were obtained, and smallest for the SJC. In addition, it was determined that women with only one swollen joint or none had significantly higher levels of subjective disease activity, and also a higher DAS28, than men. This resulted in a lower percentage of females who would be considered by DAS28 to be in remission. The authors conclude that "RA disease activity measures appear worse in women than in men, [but that] most of the gender differences ... may originate from the measures of disease activity rather than from RA disease activity itself."

This study is respectable in terms of its purpose and scope, and enviable for its patient material. Nonetheless, it is not completely clear what these data mean. Several important aspects are touched upon, but not all can be addressed entirely satisfactorily. The first key issue is whether there is a sex difference in the course of RA: do women in fact 'do worse'? On several points this study simply cannot provide the answer. The most dramatic and definitive long-term outcome in RA is mortality: many studies have shown that patients with RA die mostly of the same causes as others, but they die younger, presumably due to the long-term consequences of the disease in terms of natural immunity, cancer surveillance, atherogenesis, etc [25-27]. No cross-sectional study could ever demonstrate whether there is a difference in this outcome. To the contrary, 'left-censoring', the fact that patients who did have the worst disease might have died before being able to contribute to the cohort, could have attenuated any true difference between the sexes. The second key issue has to do with physical function in patients with RA. A sex difference in functional capacity for patients with RA has been noted previously and was also confirmed in this study, with women having more functional impairment than men, but this finding is open to different interpretations. It could be a difference in disease course, but also a difference between the ability of men and women to compensate for any functional losses in daily life (primarily due to the above-mentioned difference in muscle strength, but also in bone mineral density, skin thickness and so on). Indeed, the most widely used measure of function, the HAQ, is cross-sectionally always higher (worse) in women.

In order to settle these and related questions more definitively longitudinal studies of large cohorts of patients are going to be needed. The QUEST-RA group may be well positioned to perform such studies and we are eagerly awaiting such data. Randomized trials, which by necessity are more restrictive and of shorter duration, may also provide valuable insight into any true sex differences in the course and prognosis of RA. Ultimately, such results may impact on our understanding of autoimmune diseases as well as on their treatment, for instance, by having sex-specific treatment algorithms if outcomes can be shown to differ by sex and by medication.

Finally, the conclusion drawn by Sokka et al, that differences between the sexes are explained by the measurement of the disease rather than by the disease itself, is an interesting one, but perhaps not fully supported by their data. This touches on a somewhat sensitive issue that we recently investigated at our unit [28]. The point is this: if the 'objective' disease, that is, the amount of inflammation in the joints, is identical, but the female patient experiences this as 'more' disease, that is, more pain, more stiffness, more generalized distress, and more functional deterioration, is it then fair to say that the disease nonetheless is identical? This would reflect, in my opinion, a rather limited biological perspective. Before dwelling on that, let me clarify that we do not yet know that this is indeed the case. However, if it were true it should not come as a surprise: it is well established, for instance, that given the same noxious stimulus female experimental subjects experience a slightly higher degree of physical pain; that is, the pain threshold is lower [29-33] (this has nothing to do with the fact that women may be, according to widely held belief, 'tougher' when it comes to *coping *with pain). If that is indeed the case, should not then our assessment of how serious the disease really is also take into account the subjective dimension, the suffering imparted by the disease on the patient? It would appear to me that this is particularly true for those of us who practise (or teach the art of) medicine. After all, when we treat our patients, we do so in order to alleviate *their *suffering. And although the variability of subjective experiences and expressions compels us to be very dependent on objective data, we certainly must never forget to whom we owe our first obligation.

## Competing interests

The author declares that he has no competing interests.

## Pre-publication history

The pre-publication history for this paper can be accessed here:


